# Supplementary material for: MetaRibo-Seq measures translation in microbiomes
Source: Nat Commun. 2020 Jun 29;11:3268. doi: 10.1038/s41467-020-17081-z (PMC7324362; doi:10.1038/s41467-020-17081-z)
Supplement: Supplementary file 10 — Supplementary Data 7 [file 41467_2020_17081_MOESM10_ESM.zip › File2/Confidence_High_Taxonomy/221077_out.krona.html]

Javascript must be enabled to view this page.

members
magnitude
magnitudeUnassigned
count
unassigned
taxon
rank

221077\_out

20

20
2
superkingdom

20
976
phylum

class
200643
20

171549
20
order

family
171552
20

genus
20
838

species
20

SRS011134\_contig\_number\_contig-100\_881.283157SRS012849\_contig\_number\_16445SRS013098\_contig\_number\_12445SRS013521\_contig\_number\_5655SRS015794\_contig\_number\_7225SRS017307\_contig\_number\_30681SRS022609\_contig\_number\_28552SRS043667\_contig\_number\_481SRS045713\_contig\_number\_22316SRS046502\_contig\_number\_13654SRS047433\_contig\_number\_10290SRS049712\_contig\_number\_contig-100\_536.139856SRS049896\_contig\_number\_903SRS049995\_contig\_number\_12735SRS053356\_contig\_number\_42793SRS077194\_contig\_number\_4366SRS078176\_contig\_number\_2054SRS1041116\_contig\_number\_1140SRS105082\_contig\_number\_2682SRS143876\_contig\_number\_13632
165179
